# Systemic Signaling: A Role in Propelling Crop Yield

**DOI:** 10.3390/plants11111400

**Published:** 2022-05-25

**Authors:** Jieyu Chen, Byung-Kook Ham

**Affiliations:** 1 Global Institute for Food Security, University of Saskatchewan, Saskatoon, SK S7N 4L8, Canada; 2 Department of Biology, University of Saskatchewan, Saskatoon, SK S7N 5E2, Canada

**Keywords:** systemic signal, photosynthesis, carbon assimilation, systemic acquired acclimation, stomata movement, stomata density, photosynthate, phloem unloading, organ development, carbon/nitrogen balance

## Abstract

Food security has become a topic of great concern in many countries. Global food security depends heavily on agriculture that has access to proper resources and best practices to generate higher crop yields. Crops, as with other plants, have a variety of strategies to adapt their growth to external environments and internal needs. In plants, the distal organs are interconnected through the vascular system and intricate hierarchical signaling networks, to communicate and enhance survival within fluctuating environments. Photosynthesis and carbon allocation are fundamental to crop production and agricultural outputs. Despite tremendous progress achieved by analyzing local responses to environmental cues, and bioengineering of critical enzymatic processes, little is known about the regulatory mechanisms underlying carbon assimilation, allocation, and utilization. This review provides insights into vascular-based systemic regulation of photosynthesis and resource allocation, thereby opening the way for the engineering of source and sink activities to optimize the yield performance of major crops.

## 1. Introduction

With the acute demand for enhancing food production within currently changing agricultural environments, researchers are seeking solutions to maintain crop growth under adverse conditions. During the past century, food production has been increased through fertilizer application and field management, the breeding of elite crops [1,2], genetic engineering of plant genomes, etc. [3]. In diversified agricultural ecosystems, crop productivity is largely dependent on the photosynthetic capacity of source organs (net producer of carbohydrates) and assimilation efficiency of photosynthates in sink tissues/organs (net consumption of imported carbon sources).

Great efforts and considerable progress have been made towards understanding the molecular mechanisms and regulatory events in photosynthesis. For example, facilitated by advanced biophysical technologies, researchers have developed well-resolved crystal structures of light-harvesting systems/complexes from cyanobacteria to higher plants, offering us a solid basis for the detailed examination of light reactions that occur during photosynthesis [4,5,6,7]. Another important component that determines the enhancement of crop yield is the capacity and efficiency of plants to assimilate carbon dioxide (CO_2_) from the atmosphere. Recent breakthroughs were reported in the engineering of C_3_ plants that increased photosynthetic potential by reducing photorespiratory-associated losses [8,9].

Photosynthetic efficiency is critical to improving crop yield and feeding the growing global population [10]. Indeed, C_3_ crops (cassava, soybean, rice, etc.) benefit from their advanced photosynthetic efficiency and higher CO_2_ assimilation rates but may require a simultaneous increase in sink capacity to enhance crop yields. This is because the upregulation of carbon utilization, in harvestable sink tissues/organs, will, in turn, enhance the translocation rate of photoassimilates being delivered from the source to the sink, thereby further driving photosynthesis in the source region. Sink strength, the capacity of plants to utilize carbon in sinks, is often reinforced by bioengineering carbohydrate metabolic pathways in sink tissue/organs [11,12]. For example, invertases play important roles in sugar metabolism, which is vital for sink strength regulation in many systems. Cytosolic expression of yeast invertase can lead to a reduction in starch content in potato tubers; however, when the same enzyme was targeted in the potato apoplast, it resulted in the enlargement of tuber size and enhancement of yield [13]. In addition to the regulation of sucrose cleavage by invertases, sink strength may also be adjusted by the enzyme activities involved in the biosynthesis of storage compounds and compartmentation of sucrose in sink cells [14,15].

Studies on source production and sink assimilation alone have advanced our understanding of carbon accumulation in crops; however, knowledge about how plants transmit environmental cues from source to sink tissues to adjust biological processes in the newly established young sinks remains largely unknown. Recent discoveries about source–sink correlations have begun to provide new insights into pathways for optimizing crop growth in an ever-changing global environment [16]. Here, we provide an overview of recent findings that highlight the important roles played by systemic signals in carbon assimilation and utilization. Future prospects regarding the application potential provided by these technologies are also discussed.

## 2. Systemic Regulation of Carbon Assimilation and Allocation

### 2.1. Photosynthesis

Selective breeding programs have endowed modern crops with high efficiency in expanding leaf area and the translocation of carbon and nutrients into seeds. However, many energy conversion limitations still limit the overall efficiency of photosynthesis. Crops harvested in temperate and tropical areas can only convert 1% of the annual solar irradiance over the same land area [17]. Light and CO_2_ are two driving forces for photosynthesis. Plants respond to light intensity, light quality (spectral distribution), and CO_2_ in their habitat through various physiological and developmental adjustments. Plants’ long-distance signaling, i.e., systemic regulation of distal organs/tissues, has attracted increasing attention in terms of being an important adaptation to environmental changes. Integrative studies have indicated that light- and CO_2_-dependent, leaf-to-leaf long-distance signals mediate processes that are closely related to photosynthesis, and the underlying mechanisms have provided insight into the optimization of crop yield [18,19,20,21].

#### 2.1.1. Light-Dependent Systemic Signaling—Systemic Acquired Acclimation

Although light is the energy source for photosynthesis, excess excitation energy (EEE) can be harmful to photosynthetic organisms. When light energy exceeds the level required for CO_2_ assimilation, the redox status of the photosynthetic electron transport chain will be changed, and the generation of reactive oxygen species (ROS) will be accelerated [22,23]. ROS production can lead to direct oxidative damage to photosystem Ⅱ (PS Ⅱ) [24], and/or inhibit its repair, eventually resulting in a reduction of photosynthetic efficiency [25].

**Figure 1:**
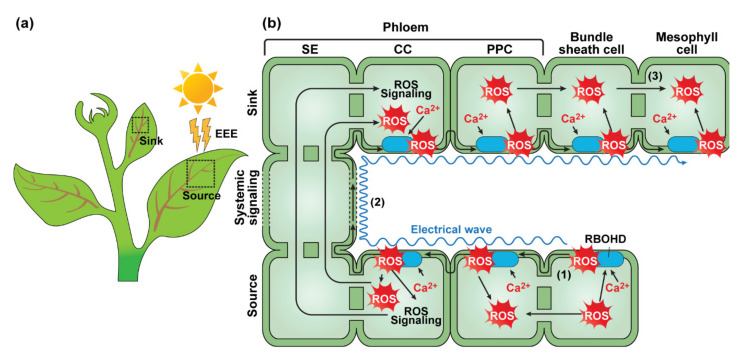
Systemic acquired acclimation (SAA) occurs through a signal network within the plant vasculature. (**a**) Schematic representation of an exposed leaf that receives excess excitation energy (EEE), and a systemic leaf, located at a sink region. (**b**) Proposed signal transduction pathways in SAA. (1) Initial burst of cellular ROS triggers the cascade of cell-to-cell ROS waves, i.e., hydrogen dioxide (H_2_O_2_) is produced by Respiratory Burst Oxidase Homolog D (RBOHD) (indicated by blue ovals) on the bundle sheath cell plasma membrane. (2) RBOHD relays and maintains an auto-propagating ROS wave along with the plant vascular bundle. Signal amplification propagates rapidly in the companion cell (CC)—sieve element (SE) complexes within the phloem, which may involve calcium ions (Ca^2+^) and electrical signals. (3) In distal unexposed leaves (systemic leaf), the auto-propagated ROS wave and phloem-transmitted signals are perceived by mesophyll cells, thereby a local defense system is triggered, and the cells within young leaf tissues are acclimated in preparation for the approaching strong EEE treatment to protect their photosynthetic efficiency. PPC, phloem parenchyma cell.

It was shown that excess light stimulates a defense system within cells of exposed leaves, in response to ROS generation [26,27,28]. Moreover, upregulation of this defense is not only observed in the treated leaves (directly exposed to excess light), but also in the systemic leaves (distal non-treated leaves). When untreated systemic leaves of the same exposed plants were subsequently exposed to excess light, these pre-acclimated leaves were more tolerant to the treatment, manifested by changes in the redox status and only a slight reduction of the pre-acclimated leaves’ PS II photosynthetic efficiency. This phenomenon was termed systemic acquired acclimation (SAA) [18,29] (Figure 1a). This SAA involves a complex network of ROS [30], calcium (Ca^2+^) [31], and electric signals [32], which are transmitted through the vasculature and induce changes in gene expression profiles, thereby establishing resilience to light stress at the whole-plant level [33].

Pioneering research on SAA demonstrated that light-induced hydrogen peroxide (H_2_O_2_) accumulation within vascular tissues of the leaf triggers systemic signal transmission and enhances resistance to excess light in Arabidopsis [18,34]. Further studies of systemic signaling under high light stress have suggested that H_2_O_2_ production, in the apoplast of bundle sheath cells, is dependent on NADPH oxidase activity, and the ROS accumulation could be enhanced upon the biosynthesis of abscisic acid (ABA) in adjacent vascular parenchyma cells [35]. Furthermore, studies based on a luciferase reporter line, under the control of the ROS-responsive *ZINK FINGER OF ARABIDOPSIS THALIANA 12* (*ZAT12*) promoter, demonstrated that excess light treatment could induce the rapid spreading of an ROS wave, via the vascular system [36,37]. This ROS wave occurred via the function of a specific NADPH oxidase isoform, RESPIRATORY BURST OXIDASE HOMOLOG D (RBOHD). This enzyme triggered and maintained an auto-propagating ROS wave along the plant axis, at a rate of 8.4 cm/min [38,39].

A calcium wave can be defined as the systemic movement of cytosolic Ca^2+^. GLUTAMATE RECEPTOR-LIKE (GLR) proteins play a pivotal role in increasing the cytosolic Ca^2+^ level to intercellularly propagate a Ca^2+^ wave [31,40]. Ectopic expression of PLASMODESMATA-LOCATED PROTEIN 5 reduced this spread of the cytosolic Ca^2+^ increase, suggesting a role of PD in the transmission of the Ca^2+^ wave [40]. Recent discoveries suggest that the correlation between ROS and Ca^2+^ waves, in terms of SAA, is associated with a core set of transcripts that are induced upon the function of RBOHD and ROS waves [41]. ROS could trigger the TWO PORE CHANNEL1 (TPC1), a vacuolar cation channel, to increase the cytosolic Ca^2+^ concentration [42]. As RBOHD is activated by Ca^2+^ [43], its activity is likely dependent upon the cytosolic Ca^2+^ level for the production of ROS in the apoplast (Figure 1b). 

An electric wave can be generated by various stimuli, e.g., temperature or wounding, and is a common systemic signaling mechanism within land plants [33]. Recent studies have proposed a correlation between increasing cytosolic Ca^2+^ levels and the propagation of an electrical signal. The membrane depolarization is followed by a peak of cytosolic Ca^2+^ in an Arabidopsis wild-type leaf, whereas in plants carrying mutant forms of GLR3.3 and GLR3.6, the calcium flux is impacted, causing attenuation of electrical signal propagation [40,44,45]. Based on the enzymatic feature of RBOHD, which is activated by the Ca^2+^ signal, an electric wave could be integrated with the ROS wave, through TPC1, which is triggered by ROS. In *Mimosa pudica* and the Venus flytrap (*Dionea muscipula*), mechanically triggered electrical signals can inhibit photosynthesis [46,47]. Thus, taken together, these findings support the notion that the integration of ROS, Ca^2+^, and electrical signals are regulated, in a sophisticated manner, by each other, and function in the rapid long-distance signaling mechanisms, during SAA, in response to excess light exposure (Figure 1b).

Interestingly, this rapid systemic signaling mechanism can also be induced by other abiotic and biotic stresses, including wounding, extreme temperatures, salinity, and pathogen attack [48]. Noticeably, combinations of these abiotic and biotic stresses frequently occur in nature or agricultural practice. As discussed above, accumulating evidence suggests that the rapid spreading of a ROS wave along the vascular system is highly correlated with the acclimation of the plant to excess light [37,41]; however, it is not sufficient to interpret the specificity of the imposed environmental cues [38,48]. Therefore, the rapid transmission of ROS signals is possibly integrated into other signaling pathways to improve the fitness of plants under high light stress [19,49,50,51,52].

Sunlight distribution incident on different leaves varies under natural conditions. Here, EEE, experienced by plants, is also determined by other environmental and developmental factors, which may lead to changes in the required amount of light energy for cellular processes. Pathogen attack, nutrient deficiency, water stress, and rapid temperature fluctuations can result in EEE, even for a light intensity suitable for plant growth under normal conditions [53,54,55,56]. The potential for generating an EEE condition is a constant challenge for land plants, and failure to dissipate or avoid EEE will result in photooxidative damage to the photosynthetic apparatus, with a commensurate dramatic decrease in photosynthetic efficiency. Therefore, it is critical to further understand the importance of SAA in nature, which would offer the potential to enhance the pre-acclimation of shaded leaves in crops to subsequent full sunlight exposure, and thus improve plant growth performance and productivity within fluctuating environmental conditions.

#### 2.1.2. CO_2_-Related Systemic Signaling—Regulation of Stomatal Movement and Density

Plants depend on photosynthetic carbon for growth and reproduction. Higher photosynthetic efficiency can be achieved by enhancing CO_2_ uptake from the atmosphere and maximizing the biochemical rate of CO_2_ fixation. However, this positive correlation, between increased CO_2_ uptake and improved photosynthetic efficiency, needs to be considered in the context of the surrounding environment, because both CO_2_ diffusion into the intercellular airspaces of leaves and water loss from plants predominantly occur through stomatal pores. In this regard, it was shown that, under drought conditions, both barley and rice increased their harvest index through the effective manipulation of tradeoffs between carbon assimilation and water loss, by reducing their stomatal densities (SD, stomata numbers per unit leaf area) [57,58].

Leaf gas exchange is largely determined by the extent of stomatal aperture and SD. The best-studied systemic signal that controls stomatal movements is the root-derived hormone, abscisic acid (ABA), which, under drought stress, promotes stomatal closure to conserve water [21,59,60]. However, ABA biosynthesis is important in leaves to enhance dehydration tolerance under long-term drought conditions [61]. A recent study proposed a mechanism by which a root-derived peptide molecule serves as a mobile signal to promote ABA biosynthesis in leaves for stomatal closure [62]. Under dehydration stress conditions, a CLAVATA3/EMBRYO-SURROUNDING REGION-related25 (CLE25) peptide is derived within the root vascular tissue and traffics into leaves, in which CLE25 is recognized by the BARELY ANY MERISTEM1 (BAM1) and BAM3 receptors to promote the expression of *NINE-CIS-EPOXYCAROTENOID DIOXYGENASE3* (*NCED3*) to increase ABA biosynthesis [62]. Thus, CLE25 systemic signaling appears to mediate ABA accumulation in leaves for stable responses to mid- and long-term drought stress.

**Figure 2:**
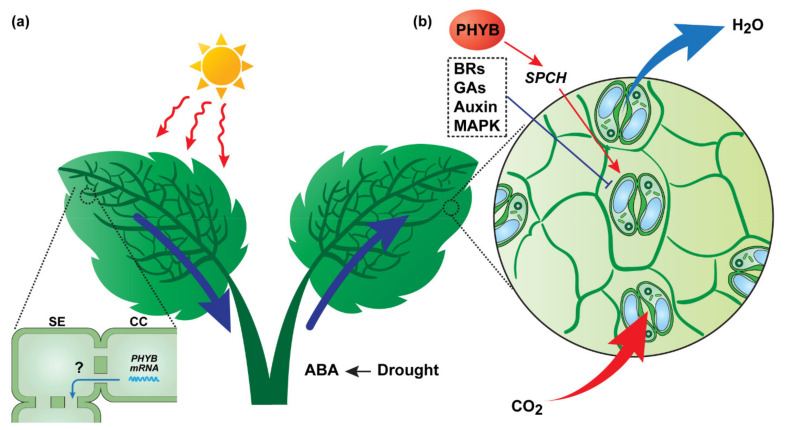
Schematic representation of long-distance signaling pathways that control stomatal movement/development. (**a**) Phytochrome B (PHYB), the red-light receptor, may exert a regulatory function on systemic control of stomatal development in the developing leaves. Under strong light conditions, mature leaf-specific expression of the *PHYB* gives rise to mobile *PHYB* transcripts that are delivered to unexposed developing leaves, where they are translated into functional protein to regulate downstream genes, e.g., SPCH, thereby relaying environmental cues (light intensity) from the exposed to developing leaves. Under drought stress, root-derived abscisic acid (ABA) acts as systemic signals to promote stomatal closure, to minimize water loss from the plant. The question mark indicates potential underlying mechanism for long-distance movement of *PHYB* mRNA, which remains to be elucidated. (**b**) Increase in CO_2_ concentration, sensed by source leaves (major photosynthetic sites), triggers a local signaling network, which initiates downstream cascade responses and communicates the changing condition to young developing leaves. The long-distance signaling may adjust the expression profiles of the genes that are involved in auxin, brassinosteroid (BR), gibberellin acid (GA), and MAPK signaling pathways.

Cytokinin and other biochemically active molecules are reported to regulate stomata sensitivity to ABA [60,63,64,65,66,67], and cytosolic alkalinization in guard cells precedes an ABA-triggered signaling cascade that may play an important role in ABA-regulated stomatal closure [68,69]. In addition, the water content in the environment (humidity) is suggested to mediate systemic regulation of stomata development. Vapor pressure differences between the leaf and atmosphere determine the transpiration rate of plants, and further, changes in the transpiration rate can modify the stomatal aperture of mature leaves and the SD of developing leaves. The ABA-deficient mutant, *aba1*, exhibited a disruption in the SD response to transpiration, which could be restored by the application of exogenous ABA to mature leaves [70]. These findings provided strong support for a model in which the response of stomatal movement/development to humidity, through the regulation of the transpiration rate and stomatal conductance, involves the ABA signaling pathway [71] (Figure 2).

Light, as the driving force of photosynthetic processes, plays a critical role in stomatal differentiation. The systemic regulation of light intensity on stomatal development of young developing leaves has been observed in Arabidopsis [72,73], tobacco [74], and sorghum [75], establishing that the shading of mature/expanded leaves can lead to decreased SD in younger leaves. Here, the red-light photoreceptors, phytochrome B (PHYB), may mediate this light-dependent systemic regulation of leaf stomatal characteristics [76]. The complementation of a *phyB* knockout mutant via the phloem-specific expression of *PHYB* and the induction of *PHYB* in mature leaves can restore stomatal development in untreated developing leaves under high light [77]. Additionally, it was observed that, compared to the wild-type control, the *phyB* knockout mutant was defective in the systemic control of key regulators, such as the basic-helix-loop-helix (bHLH) transcription factor, *SPEECHLESS*, that acts in stomatal development [77].

Despite a potential role for PHYB in the systemic signaling of light-mediated stomatal development, it may not be able to pass through PD, connecting phloem companion cells to their neighboring sieve elements, due to the molecular weight of PHYB (129 kDa) being above the PD size exclusion limit (SEL) [78]. Thus, the PHYB protein may not be able to enter the sieve tube system for long-distance transport to developing leaves being exposed to high light conditions. However, cell-to-cell mobility of the *PHYB* mRNA has been proposed, based on mobile transcriptomics analysis performed on heterografted *Arabidopsis* plants [79]. Therefore, the *PHYB* transcript may well function to transmit a light-dependent systemic signal to developing leaves (Figure 2). Here, further experiments are required to elucidate the underlying mechanism.

Phytohormones have been proposed as systemic signals involved in CO_2_-dependent stomatal development. Coupe et al. conducted transcriptomic analysis on untreated developing and mature leaves, under time-course treatments with elevated CO_2_ and shading [73]. In their study, 183 genes were identified from both treated mature leaves and untreated developing leaves in response to environmental cues. Based on gene ontology classification, these responsive genes are enriched in the auxin signaling pathway, MAPK cascades, and the brassinosteroid and gibberellin signaling components, suggesting that, in developing leaves, phytohormones may function as systemic signaling reagents that mediate CO_2_-dependent stomatal development (Figure 2b). This finding is in accordance with recent discoveries about how these phytohormone signaling pathways integrate into CO_2_-dependent stomatal development [80,81]. However, the underlying mechanism(s) by which the source leaf and young developing leaf communicate with each other, through long-distance hormonal signals to modulate CO_2_ fixation under optimal growth or stress conditions, still awaits further studies.

Stomata on mature leaves may act as stress signal-sensing and transduction centers by adjusting their movement. Although mechanisms of stomatal aperture response are well characterized, the pathways by which mature stomata relay environmental cues to developing leaves and ultimately regulate stomatal density are not. Environmental change, i.e., elevating atmosphere CO_2_, has impacted crop photosynthetic profiles worldwide, and thus, developing an understanding of the systemic signaling in stomatal short- and long-term responses will be critical to balancing photosynthetic efficiency (carbon assimilation) and water efficiency (transpiration rate) to achieve improved agricultural output under limited water supplies [82].

### 2.2. Assimilate Loading and Partitioning

The plant vascular system consists of xylem, phloem, and other integral tissues [83]. Xylem functions as an efficient water and mineral nutrient transport system, from root to shoot, and provides essential mechanical strength for the plant body. In mature phloem, enucleate sieve elements (SEs) are sustained by symplasmically interconnected companion cells (CCs), thereby forming a system of sieve element–companion cell (SE-CC) complexes. These complexes connect to surrounding cells via apoplasmic or symplasmic methods [84]. Further, SEs are arranged end-to-end, giving rise to the structure referred to as sieve tubes, which function as the conduit through which the phloem translocation stream moves to transport photosynthates from the source to sink areas of the plant, as well as in the delivery of information molecules to distal organ/tissue [83,85].

Photosynthates flow between source leaves (net production of photoassimilates), and sink organ/tissues (net consumption of resource) can be characterized by three physiological processes in succession: (1) Photosynthates are loaded into collection phloem in minor veins of source leaves; (2) long-distance delivery of these loaded materials to distal sink organs through transport phloem; and (3) photosynthate (generally sucrose) exits from release phloem into the surrounding tissues for utilization or storage. Great efforts have been made to manipulate metabolic enzymes and transporters that are involved in photosynthate assimilation within the source and/or sink organs’ development, endeavoring to improve agricultural output under fluctuating environments [86,87,88,89].

Increasing evidence has pointed out that source–sink interactions play a critical role in regulating plant growth and reproduction, and hence, crop yield [12,90,91]. Moreover, Ham and Lucas [78] have stated that local environmental inputs, as well as global integrators, can adjust or even override the internal metabolic and developmental needs of plants. Here, we will highlight a few examples to discuss recent studies on the systemic regulation of source allocation by phloem-borne long-distance signals. 

#### 2.2.1. SlCyp1, a Mobile Protein Required for Auxin-Mediated Lateral Root Development

Within meristematic sink tissues/organs, phytohormones play a critical role in the regulation of cell division and elongation, and hence, developmental sink demands. For example, auxin is an essential molecular player for root system architecture regulation, being highly involved in primary root elongation and lateral root formation [92,93]. Biosynthesis, perception, signaling, and polar transport of auxin are required for normal lateral root development [94]. SlCyp1, a tomato gene that encodes a peptidyl-prolyl cis-trans isomerase, has been reported to mediate lateral root development through alteration in the localization of PIN-FORMED auxin efflux transporters [95]. Despite the detection of the Cyp1 protein in phloem exudates collected from various species [96,97,98], the role of Cyp1, as a phloem-borne systemic signal, in the regulation of root development has only recently been deciphered [93,97,98].

**Figure 3:**
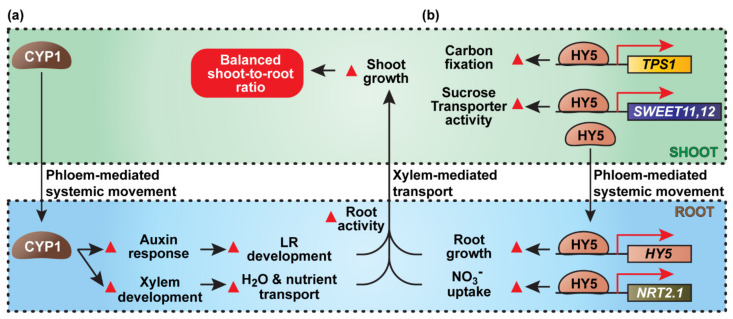
Light promotes root growth and nutrient absorption. (**a**) CYP1 is a phloem mobile protein that regulates shoot-to-root homeostasis. Light intensity determines the expression level of *CYP1* in source leaves. Shoot-derived CYP1 protein accumulates in the roots, leading to activation of the auxin responses, thereby enhancing lateral root formation. Phloem-mediated systemic regulation, through this CYP1 pathway, is also involved in xylem development, which is critical for transport of H_2_O and mineral nutrients from roots to shoots. (**b**) Arabidopsis ELONGATED HYPOCOTYL5 (HY5), a key positive regulator of light signaling, is a phloem mobile transcription factor that traffics from shoot to root. In the aboveground tissue, HY5 binds to the promoters of *TPS1, SWEET11,* and *SWEET12* to regulate carbon fixation and phloem-mediated transport of photoassimilates. Mobile HY5, derived from shoots, binds the promoter regions of *HY5* and *NRT2.1* genes in the root to promote root growth and nitrate uptake. Both shoot-derived CYP1 and HY5 can lead to enhanced root activity to improve shoot growth. These long-distance signaling agents also contribute to balancing the plant’s shoot-to-root ratio. Red darts indicate up-regulation of described responses.

The pleiotropic phenotypes of the *diageotropica* (*dgt*) mutant, which carries a point mutation on SlCyp1, result in a defect in auxin response pathways, and hence, reprogramming of the transcription profile of the mutant root system [95,99]. Experiments using heterografting techniques, employed between wild-type tomato scion and the *dgt* mutant rootstock, showed that the graft-transmissible SlCyp1 signal could restore xylem differentiation, root response to auxin treatment, and lateral root formation in the mutant rootstock [95,99,100] (Figure 3a).

Another noteworthy finding from this work is that the shoot perception of light intensity plays an important role in adjusting SlCyp1 protein abundance in mature source leaves and its movement through the phloem translocation stream to the root, which then further mediates systemic regulation on the root system architecture [99,101]. Although the molecular mechanism by which SlCYP1 participates in the auxin signaling network remains to be elucidated, the SlCyp1 is an important illustration of the function of a phloem-borne mobile signal that serves to integrate light intensities, incident on photosynthetic source leaves, and root system architecture underground in the soil via a positive feed-forward intervention through auxin-mediated pathways.

#### 2.2.2. Switching of Unloading Pathways by an Interaction between an FT Orthologue and SWEET

Photoassimilates are delivered through the phloem to heterotrophic tissues/organs and unloaded for multiple uses. Sucrose unloading may occur either symplasmically through plasmodesmata interconnecting the SE-CC complexes with the surrounding cells or apoplasmically (across cell walls) via a combination of sucrose transporters [102,103]. In some expansion/storage tissues/organs, both unloading pathways may function in sequence, and sometimes these two processes may shift in response to developmental and environmental cues. A well-studied case in this regard is the transition from apoplasmic to symplasmic unloading in potato during the early stage of tuberization [104].

The apoplasmic unloading pathway dominates during the development of the potato’s underground lateral shoots (termed stolons). Here, sucrose translocated through sieve tubes is released (unloaded) into the apoplasm by Sugars Will Eventually Be Exported Transporters (SWEETs) [105], followed by its uptake into sink cells through the action of sucrose/H^+^ symporters. Alternatively, this sucrose can be hydrolyzed by cell-wall-bound invertases, yielding hexoses that are then taken up into the surrounding cells via hexose transport proteins. Under short-day photoperiodic conditions, the potato orthologue to the Arabidopsis florigen, FT, Solanum tuberosum SELF-PRUNING (StSP) 6A, moves from the source leaves to the stolons where it promotes stolons to form tubers [106]. Tuberization is characterized by the switch from an apoplasmic to symplasmic unloading pathway, which then leads to an increase in sucrose unloading efficiency and enhanced tuber growth. Although this physiological process has been characterized, the underlying mechanism of the switch is yet to be elucidated.

**Figure 4:**
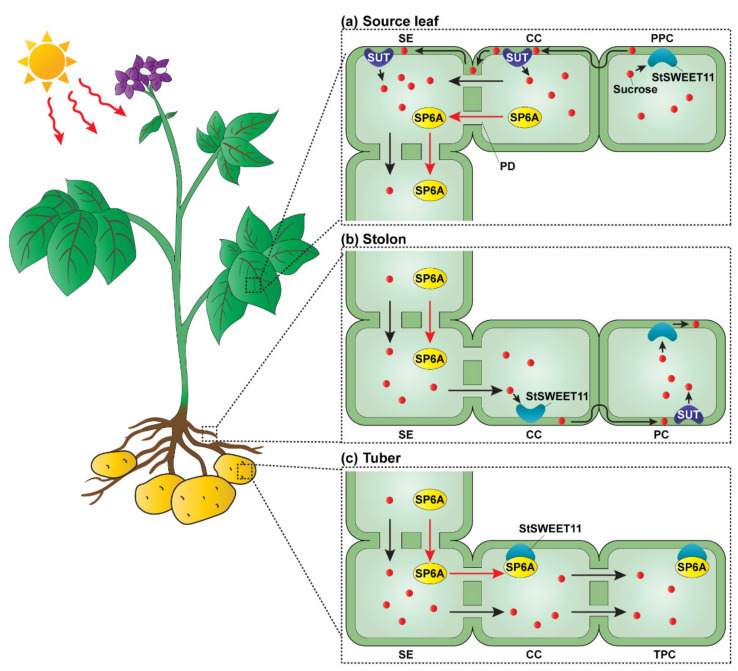
Mobile SP6A mediates StSWEET11 function in potato tuberization. (**a**) Sucrose is transported from phloem parenchyma cells (PPC) to companion cells (CC) in the source leaves, using the apoplasmic pathway. Sucrose transporters (SUTs) load sucrose into sieve elements (SEs) and CCs to establish a high concentration in the phloem. Sucrose also can move symplasmically from CCs to SEs through plasmodesmata (PD). High levels of sucrose promote *SP6A* expression in CCs of source leaves; PD-mediated intercellular movement of SP6A allows its long-distance trafficking through the phloem. (**b**) In the stolon, sucrose moves symplasmically from SEs to CCs where it is then unloaded, via the StSWEET11 permease, into the apoplasm. Sucrose is retrieved from the apoplast of parenchyma cells (PCs) by the SUTs. (**c**) Under tuberization, shoot-derived SP6A is unloaded into the stolon CCs where it interacts with StSWEET11 permeases to block apoplasmic sucrose transport between CCs and tuber parenchyma cells (TPCs); thereby, sucrose moves symplasmically from CCs to TPCs. The transition of sucrose unloading pathway in TPCs, from apoplasmic to symplasmic, facilitates tuberization process under high availability of sucrose. Red small round circles indicate sucrose molecules.

In a recent finding, researchers established a correlation between photoperiod properties perceived by source leaves, and the sugar unloading pathway in stolon tissues, reflecting the role of source–sink communication during potato tuberization [107]. Additional insight into the factors that induce tuberization was provided by a study in which a potato orthologue of AtSWEET11 was identified as a candidate linking StSP6A and sucrose induction of tuber formation. Here, a direct protein interaction was shown at the plasma membrane between the carboxyl terminus of StSWEET11 and StSP6A. Furthermore, the heterologous transformation of both proteins confirmed that this interaction with StSP6A inhibited the transport activity of StSWEET11. In terms of a potential mechanism, the measurement of the sugar levels in the apoplastic fluid and protoplasts derived from stems of different genetic materials suggests that this in vivo interaction of StSP6A with StSWEET11 may block sucrose release (apoplasmic unloading) from the phloem, which then initiates sucrose unloading through the symplasmic pathway (Figure 4).

The change from apoplasmic to symplasmic unloading is followed by the induction of tuber formation. This switch reflects a transition from an energy mode to a storage mode of metabolism. These new findings on the correlation between photoperiodic regulation and sucrose transport, in potato, provide important insight into the complex mechanism underlying the biological processes associated with photosynthate allocation via the phloem within the body of the plant. In this regard, furthering our understanding of the processes involved in the fine-tuning of the molecular functions involving StSP6A will be of considerable importance in the engineering of source–sink coordination to improve crop yields.

#### 2.2.3. Shoot-to-Root Transmission of HY5 Mediates C/N Balance in Response to Light Conditions

The efficiency of photosynthate assimilation and allocation is critical for crop yield. However, crop productivity is frequently limited by nitrogen (N) availability within agricultural soils, as N plays a pivotal role in the maintenance of the photosynthetic apparatus, as well as regulating sink strength by adjusting plant growth and development. On the other hand, the N resources in soils, such as nitrate and ammonium, are absorbed by plant roots and assimilated for diverse uses through processes requiring reductants, energy, and carbon skeletons generated from photosynthesis. Therefore, N and carbon (C) metabolism are tightly linked in a wide range of biological processes within plants [108]. 

Pioneering studies have revealed a relationship between environmental light conditions and C metabolites on N acquisition and metabolism [108,109,110,111]. Subsequent studies have established the presence of an extremely complex regulatory network that exerts control over C and N interaction within the plant. The availability of inorganic nutrients, metabolites, and gene products serves as important inputs to regulate key enzymes and transporters, at both the transcriptional and translational levels, which mediate the coordination of C and N assimilation [112].

Studies on the photomorphogenic-related transcription factor, ELONGATED HYPOCOTYL5 (HY5), have significantly advanced our understanding of C–N interactions at the whole-plant level with regard to the role of vascular-based signaling [113]. Here, an Arabidopsis null mutant of HY5 was characterized as deficient in light-promoted root extension and lateral root proliferation. Furthermore, shoot-illumination promotion of nitrate uptake by NRT2.1 was reduced in such *hy5* mutants, revealing that photosynthetic performance is coordinated with nitrate absorption in an HY5-dependent manner. To explore the mechanism underlying these findings, transgenic lines were developed that expressed HY5 under the control of shoot- or phloem-specific promoters. Grafting of the *hy5* mutant rootstocks onto scions of these transgenic lines restored root growth and nitrate uptake in these chimeric plants. Further, grafting assays provided evidence that HY5 functions as a phloem-mobile protein, moving from the shoot to the root where it activates root *HY5* expression via an auto-regulatory feedback loop, which likely enhances light-regulated root growth and nitrate absorption (Figure 3b).

Based on the transcriptional analysis, HY5 mediates carbon fixation and allocation, through direct binding onto the promoters of C metabolism-related genes. Auto-activation of *HY5* expression in the root was also proven to be critical for *NRT2.1* upregulation and nitrate uptake capacity. Although it remains unclear as to the underlying mechanism by which light and sugar potentiate HY5-dependent nitrate absorption, this study provides insight into an important pathway of an intertwined C/N interaction network linking N-associated metabolism to organ growth and development, as in previous reports [114], and added a new dimension to the biological processes regulated by long-distance mobile proteins, next to CYP1, FT, and StSP6A [99,106,107,115,116].

## 3. Concluding Remarks and Future Prospects

The ever-increasing demand for food and the simultaneous deterioration of agricultural environments are exacerbating the need to improve crop yield performance. To address this problem, significant efforts are being made to enhance crop production under adverse growth conditions.

Numerous studies have established that enhanced photosynthesis can improve crop yield potential [10]. Many of these breeding efforts have sought to improve aspects such as source organ/tissue capacity, including optimizing light capture by changing leaf morphology or light reaction efficiency [86,117], bypassing photorespiration to enhance carbon assimilation and growth [118], modifying rates of sucrose synthesis and sucrose signaling networks [119,120,121], introducing the C_4_ metabolic pathway into C_3_ plants [122], and so forth. These advances were primarily based on work focused on local (organ or cell-type specific) responses. Studies on the roles played by systemic signaling in the regulation of adaptive biological processes to biotic and abiotic stresses are still in their infancy.

Recently, advanced genomics technologies led to studies that highlighted the need to further explore the broad spectra of mobile signals and their impact on systemic signaling networks [78,123,124,125,126]. Moreover, several proteomics studies revealed the presence of up to thousands of proteins within the phloem exudate by using optimized sample collection techniques and highly sensitive mass spectrometry technology. These detected peptides and proteins, which are loaded into the sieve tube system, may function as systemic signals that regulate biological processes in distantly located sink tissues/organs [97,98,127,128]. Although significant progress has been made in our understanding of the components present in phloem exudates, under normal and stressed growth conditions, the challenge remains to explore the role of low-abundance phloem-borne proteins in plant development and stress-response signaling pathways. Further improvements to existing proteomics techniques may aid in the discovery and characterization of such low-abundance proteins [129].

Light capture and carbon assimilation capacity are highly correlated with agricultural productivity. Despite abundant evidence of local signaling networks regulating photosynthesis, studies on long-distance signals, potentially involved in orchestrating crop yield performance, are sorely needed. In our review, we assessed advances made in understanding the mechanisms by which systemic (long-distance) signaling adapts young leaves to fluctuating environmental parameters such as light intensity, CO_2_ levels, and humidity; we also analyzed three examples of mobile proteins, mediating biological processes in both source and sink regions of the plant, to impart information to distal tissues/organs to facilitate plant resilience to prevailing environments.

In Table 1, we also summarize additional systemic signals reported to be involved in carbon assimilation and allocation. Clearly, currently available evidence offers support for the notion that further research, aimed at identifying and characterizing mobile molecular players, in conjunction with cutting-edge gene-editing technology [130], will open doors to further improving crop plants. Various genetic, genomic, and epigenetic technologies could be used to engineer functionally mobile signals for manipulating sugar transport, carbon partitioning, and source and/or sink metabolism to modify carbon utilization within specific tissues, in order to enhance crop yield potential.

## Figures and Tables

**Table 1:** Systemic regulatory signals involved in carbon assimilation and allocation.

Signal	Species	Characteristics	Reference
Carbon Assimilation			
ROS, electrical signal and calcium	Arabidopsis	Systemic signals transmitted from excess light-exposed leaves, to untreated leaves, initiate SAA and protect the photosynthetic efficiency of young developing leaves.	[18,29]
ABA	Arabidopsis	A root-derived hormone that promotes stomatal closure, controlling gas exchange between mesophyll cells and the external environment.	[21,59,60]
*PHYB*	Arabidopsis	A non-cell-autonomous *PHYB* mRNA may function as a light-mediated systemic signal to regulate stomatal development within developing leaves.	[76,77]
Auxin, MAPK, brassinosteroid, gibberellin acid	Arabidopsis	Hormones that may function as systemic signals involved in CO_2_-dependent stomatal development.	[73]
**Carbon allocation**			
SlCyp1	tomato	A phloem-borne systemic signal that mediates root development, in response to light intensity perceived by the shoot.	[95,99,100,101]
SP6A	potato	A phloem-mobile tuberigen promotes tuberization under short-day conditions.	[106,107]
HY5	Arabidopsis	A phloem-mobile transcription factor that mediates in light-promoted root extension and nitrate uptake.	[113]
*S. tuberosum BEL1-LIKE HOMEODOMAIN PROTEIN 5* (*StBEL5*)	potato	Graft-transmissible mRNA transcripts move into the root, under short-day conditions, to enhance tuber production.	[131]
trehalose 6-phosphate (T6P)	maize	A potential regulator of whole-plant resource allocation for crop yield improvement.	[132]
